# PNGSeqR: An R Package for Rapid Candidate Gene Selection through Pooled Next-Generation Sequencing

**DOI:** 10.3390/plants11141821

**Published:** 2022-07-11

**Authors:** Sihan Zhen, Hongwei Zhang, Yuxin Xie, Song Zhang, Yan Chen, Riliang Gu, Sanzhen Liu, Xuemei Du, Junjie Fu

**Affiliations:** 1 Seed Science and Technology Research Center, Beijing Innovation Center for Seed Technology (MOA), Beijing Key Laboratory for Crop Genetic Improvement, College of Agronomy and Biotechnology, China Agricultural University, Beijing 100193, China; smilejeen@163.com (S.Z.); zhangsong0115@163.com (S.Z.); rilianggu@cau.edu.cn (R.G.); 2 Institute of Crop Science, Chinese Academy of Agricultural Sciences, Beijing 100081, China; zhanghongwei@caas.cn (H.Z.); xieyuxin@caas.cn (Y.X.); chenyhj@126.com (Y.C.); 3 Hainan Yazhou Bay Seed Laboratory, Sanya 572025, China; 4 National Nanfan Research Institute (Sanya), Chinese Academy of Agricultural Sciences, Sanya 572024, China; 5 Department of Plant Pathology, Kansas State University, Manhattan, KS 66506, USA; liu3zhen@ksu.edu

**Keywords:** bulk segregate analysis (BSA), R package, next-generation sequencing (NGS), algorithm

## Abstract

Although bulked segregant analysis (BSA) has been used extensively in genetic mapping, user-friendly tools which can integrate current algorithms for researchers with no background in bioinformatics are scarce. To address this issue, we developed an R package, PNGSeqR, which takes single-nucleotide polymorphism (SNP) markers from next-generation sequencing (NGS) data in variant call format (VCF) as the input file, provides four BSA algorithms to indicate the magnitude of genome-wide signals, and rapidly defines the candidate region through the permutation test and fractile quantile. Users can choose the analysis methods according to their data and experimental design. In addition, it also supports differential expression gene analysis (DEG) and gene ontology analysis (GO) to prioritize the target gene. Once the analysis is completed, the plots can conveniently be exported.

## 1. Introduction

Mapping genomic regions is often the first step for the characterization of both qualitative and quantitative traits. Since the early 1990s, bulked segregant analysis (BSA) has been widely used for genetic mapping [1,2]. BSA maps genetic loci using a wide variety of markers [3,4,5], including the single-nucleotide polymorphism (SNP) marker which stands out due to its ability to combine BSA with next-generation sequencing (NGS) technology [6].

The NGS-BSA procedure is typically performed based on two bulks of segregants, which includes extreme phenotypic individuals selected from a segregation population. If a gene does not control the target trait, its alleles will be randomly selected in the two bulks. Otherwise, its alleles will be enriched in each bulk. Quantification of allelic frequencies of genome-wide markers enables the evaluation of the genetic linkage between causal loci and genetic markers. Although the real allele frequencies are similar in closely linked locations, these can be affected by random noise generated by the variation in sequencing read coverage. Smoothing of discontinuous variables was proposed to reduce the noise and improve the accuracy of the result [7,8,9].

According to this basic principle, several BSA algorithms have been developed, such as G-test [7], empirical Bayes [10], ΔSNP [11], and Euclidean distance (ED) [8]. The algorithms are developed based on different sequencing data and are suitable for different situations. Additionally, these algorithms are based on various platforms and different computer languages, and different algorithms have different threshold standards. It is not convenient for researchers with little background in bioinformatics to explore BSA with multiple algorithms [12].

RNA sequencing (RNA-seq) takes fewer reads to achieve a greater read depth owning to the fact that the transcriptome is much smaller than the genome [13,14]. RNA-seq could not only recall genetic markers but could also quantify the transcript levels for further analysis, such as differential expression [13,15,16], making it a cost-effective alternative for NGS-BSA [8]. Comparing the expression profiling between mutant and wildtype lines can produce candidate genes associated with the target trait [17,18,19]. Enrichment analysis of differentially expressed genes can reveal the biological processes related to the trait [17,20].

In this study, we developed the PNGSeqR package to help researchers perform genetic mapping conveniently through R language (https://www.r-project.org/, accessed on 31 March 2021), which has the advantage of free, open sources, and multi-system compatibility [21]. The PNGSeqR package allows users to conduct multiple types of analysis by choosing different functions and parameters.

## 2. Results

PNGSeqR was developed based on R version 4.0.0 and depends on several packages, such as vcfR, dplyr, tidyverse, readr, locfit, DESeq2, ggplot2, topGO, and rtracklayer [21,22,23,24,25,26,27,28,29]. The software is freely available on GitHub (https://github.com/smilejeen/PNGseqR, accessed on 30 June 2022) and can be easily installed for use at the command line in any R-based development environment, such as RStudio. Example data and a complete user manual for its various features and functions have also been made available on GitHub (https://github.com/smilejeen/PNGseqR, accessed on 30 June 2022).

**Figure 1:**
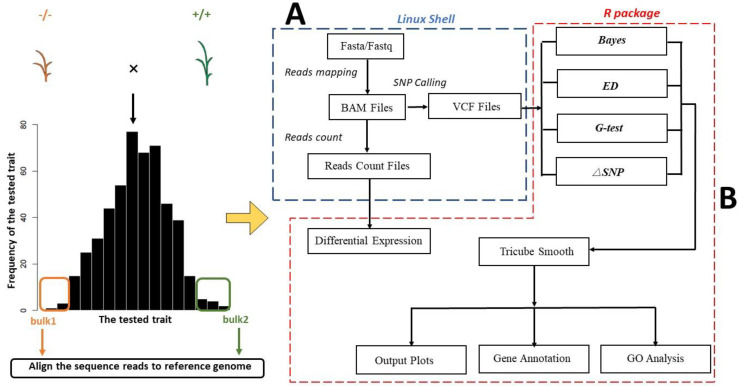
Flow chart for using R package: (**A**) The script flow running on the Linux shell system. This script will produce the VCF file, which includes NGS-SNP information, as a PNGSeqR input file. (**B**) The main statistics and functions in PNGSeqR.

PNGSeqR provides users with a straightforward pipeline with multiple functions, including (Figure 1): (1) importing SNP data in variant call format (VCF) from the Genome Analysis Toolkit (GATK) (https://github.com/broadinstitute/gatk/releases, accessed on 14 May 2019), (2) filtering SNPs that may affect the accuracy of analysis based on the quality of SNPs, (3) carrying out BSA according to the users’ experimental design, (4) identifying the candidate regions and producing the analysis results in the form of scatter plots and/or line plots, (5) performing differential expression gene analysis (DEG) and gene ontology analysis (GO) according to users’ needs, and (6) extracting the genes in the candidate region and prioritizing candidate genes based on the results of DEG and GO analysis. For users’ convenience, we provide shell command lines to create the VCF and read count files required for PNGSeqR (Supplementary Implementation).

Functions in PNGseqR can be classified into three function models. The first model is used to handle the VCF file, which can be converted into a data frame through “vcf2table()” (Table S1). The rows of the data frame show SNP information, including SNP positions, read count, and quality control information (GQ) of the reference and alternative alleles of both bulks. The number of total SNPs will be printed when the procedure is over. To reduce noise, the “BSAfilter()” function offers options for filtering based on total read depth, read depth of each bulk, the absolute difference in read depths between the two bulks, and genotype quality score (Table S1). The number of filtered and remaining SNPs will be reported after running the “BSA_filter()” function.

The second model is designed for BSA and visualization (Table S1). This model provides four functions to calculate four BSA statistics, including “DeltaSNP()”, “Bayesian()”, “ED()”, and “Gprime()”. The parameters are different for different functions. For example, the length of the reference genome and bulk size should be provided in “Bayesian()”. All functions need to set the width of the sliding window for tricube-smoothing the statistics, which reduces noisy signals. For each function, users can define candidate regions based on the quantiles and *p*-values. The “plot_BSA()” function is designed to plot the analysis results. For each statistic, a scatter plot for the unsmoothed data and a line plot for the tricube-smoothed data can be exported. If users set the threshold by inputting a quantile or a *p*-value, a red or blue line will be added on the plots, enabling the visualization of the candidate regions. The “chromlist” parameter allows users to choose which chromosome will be plotted.

The last model is designed for the identification of differentially expressed genes and performing gene annotation analysis (Table S1). “DEG_analysis()” needs to input the reads count file, and to set the number of biological replicates. Users can use “sig.level” and “exp.fold” to set the threshold for identifying differentially expressed genes. The gene list produced by “DEG_analysis()” can be used for GO analysis using the “GO_analysis()” function. Users need to provide a file containing the relationship between GO terms and the genes of the species. Several parameters, such as the types of GO analysis, methods for testing significance, and top GO terms, can be used according to the users’ needs. The parameter “plot” is optional in both “DEG_analysis()” and “GO_analysis()” functions. “DEG_analysis()” can export a volcano plot showing differentially expressed genes, and “GO_analysis()” can export the acyclic plot, bubble plot, or histogram showing the enriched GO terms. The function “resultanno()” is designed to rapidly prioritize the target genes in the candidate regions. To run “resultanno()”, users should input the position of the candidate regions identified by BSA and the annotation file in GTF format. Users can choose to input the list of differentially expressed genes in the candidate region. Table S1 shows the complete list of arguments that can be used in PNGSeqR.

We used three examples to demonstrate the performance of PNGSeqR and highlight some of the many options in this software. In example 1, a maize small-kernel mutant was crossed with a wildtype line to form an F2 population. The RNA of the mutant and wildtype samples were bulked and sequenced, and the candidate region on maize chromosome 8 was detected using multiple algorithms in PNGSeqR. Further analysis identified the DEGs in the candidate region and biological pathways that are related to the genes in the candidate. In example 2, multiple BSA algorithms were used to map the quantitative trait loci (QTLs), controlling cold tolerance of rice seedlings using sequencing data of DNA pools. In example 3, we used a recombinant inbred line (RIL) population to demonstrate how PNGSeqR can map QTLs in permanent populations.

### 2.1. Example 1—Prioritizing the Candidate Gene Controlling Quality Trait by Using RNA-Seq Data

We tested the ability of PNGSeqR to reproduce a published result of our laboratory. This work characterizes a maize mutant with defects in kernel development and clones *ZmECR1*, which encodes the RUB activating enzyme E1 subunit [20]. Raw RNA-seq data were downloaded from NCBI (accession number: PRJNA699154) and aligned to the Zea_mays.AGPv4.32 genome, and the VCF file containing SNPs and the read count file were obtained by running the shell script (Supplementary Materials). A total of 112,719 SNPs were obtained, and SNP filtering produced 79,917 SNPs, which will be used for performing BSA.

**Figure 2:**
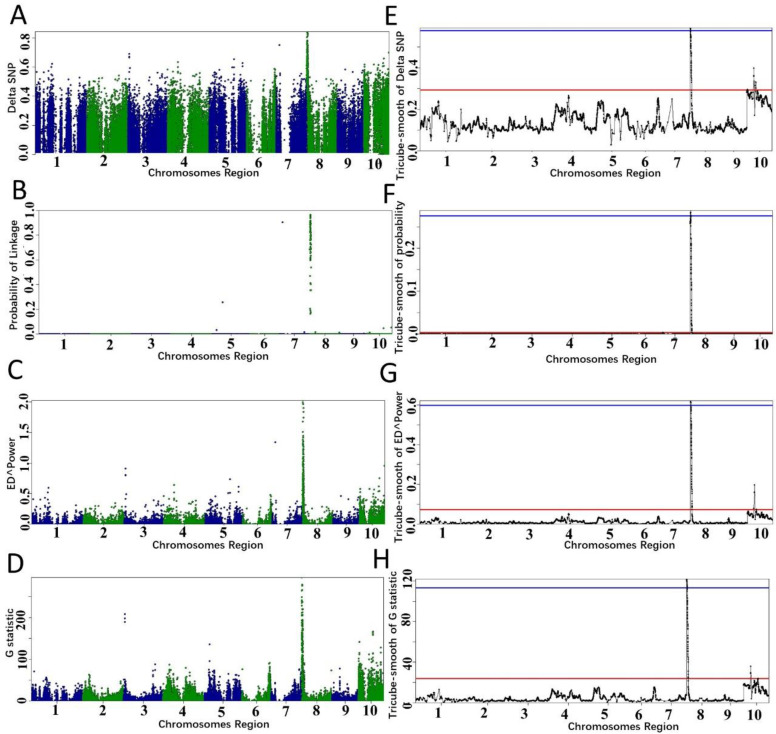
Candidate region identified by different algorithms in PNGseqR for maize small-kernel mutant. The “plot_BSA()” function was used to draw the plot with a 5 Mb sliding window. (**A**–**D**) The scatter plots were exported from the BSA results, and the algorithms were ΔSNP, empirical Bayes, ED, and G-test, respectively. (**E**–**H**) The tricube-smoothed values of the corresponding statistics. The red line is the threshold set by the >99.5% quantile, and the blue line is the threshold set by *p*-values < 0.001.

We used all algorithms for analyzing the RNA-seq data and used the analysis result to determine the threshold. All algorithms confirmed that the candidate region is on chromosome 8 (Figure 2), and the candidate regions varied with the variations of the algorithms and the threshold values (Table 1). When *p*-value < 0.01, the candidate region selected by different algorithms was 0.08–11.28 Mb, which is the same as the candidate region identified by setting the fractile quantile > 99%. When the fractile quantile was set to > 99.9%, *ZmECR1* was still in the candidate regions identified by either algorithm. When *p*-value < 0.001, *ZmECR1* was also in the candidate regions identified by two algorithms (empirical Bayesian and Euclidean distance), indicating that a stringent threshold may exclude the candidate gene from the candidate region.

**Figure 3:**
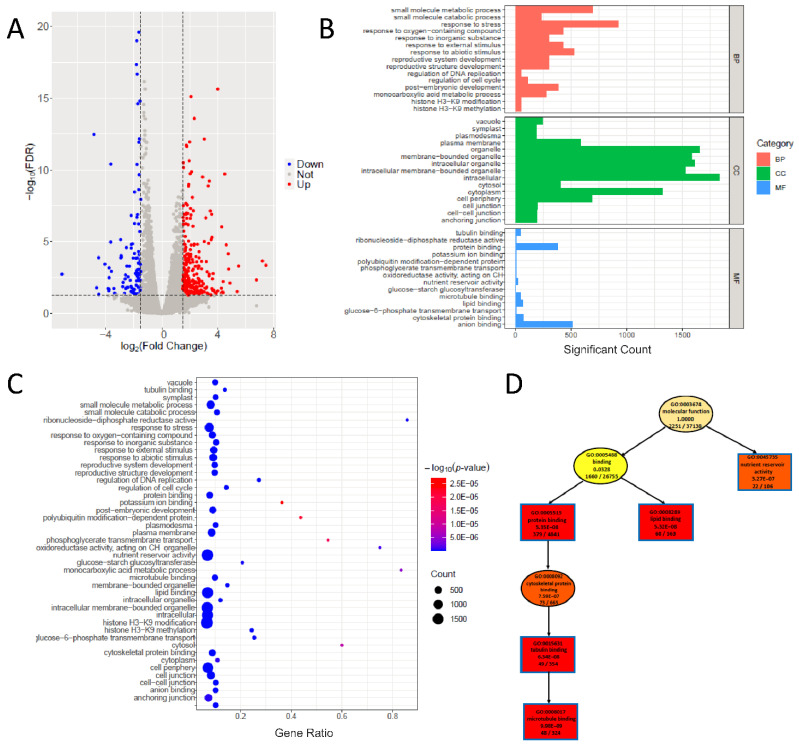
Differential expression analysis and GO analysis for the maize small-kernel mutant. (**A**) The volcano plot shows the DEGs for the maize small-kernel mutant, and the analysis was performed by using “DEG_analysis()”. (**B**) The histogram shows the result of GO analysis of differentially expressed genes for the maize small-kernel mutant, and the analysis was performed by using “GO_analysis()”. The top 20 terms of each mode (“BP”, “CC”, “MF”) are shown in this plot. (**C**) A part of the directed acyclic plot drawn using the “MF” mode of “GO_analysis()”. (**D**) Bubble plot shows the result of GO analysis, and the GO terms are the same as in (**B**).

DEGs were identified by setting the criteria as (|log_2_foldchange| > 0.5 and FDR < 0.05), and the volcano plot can be exported by using the “DEG_analysis()” function. Among the 2408 DEGs, 1130 were upregulated and 1278 were downregulated (Figure 3A; Table S2). These numbers of DEGs are larger than those reported by Chen et al. [20], which may be related to the different maize reference genome used in this study. Through inputting the DEGs and GO terms (Tables S2 and S3), we can perform GO analysis by using the “GO_analysis()” function in PNGSeqR. We found that GO terms were mainly enriched in the cell cycle, nutrient reservoir activity, structure of cytoskeleton, DNA replication, and glucose−starch metabolic process (Figure 3B–D). By using the “result_anno()” function, 92 genes in the candidate region under *p*-value < 0.001 (0.08–3.42 Mb) (Table S4) were annotated, including 7 DEGs that were identified based on the standard of |log_2_foldchange| > 1.5 and FDR < 0.05 (Table 2). As expected, the gene *ZmECR1* was one of the DEGs in this region.

These results demonstrate that the algorithms in PNGSeqR can accurately identify the candidate region and select the candidate genes by combining the BSA results with DEG and GO analysis.

### 2.2. Example 2—Mapping QTLs by Using DNA-Seq Data

**Figure 4:**
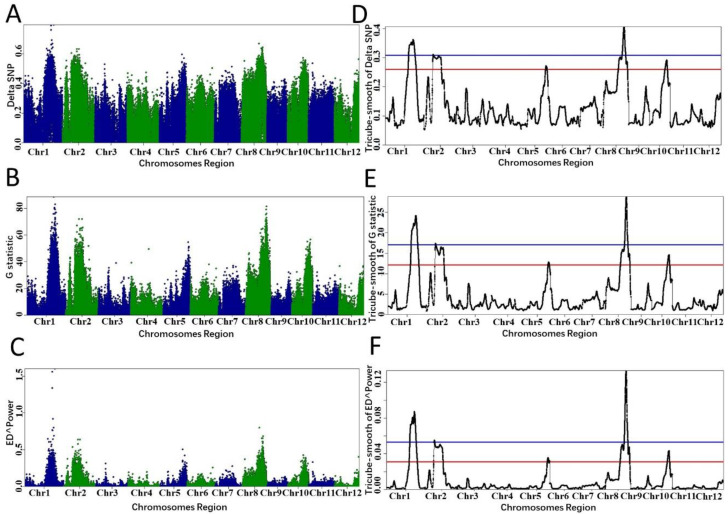
QTLs identified by different algorithms in PNGseqR for rice seedling cold tolerance. Note: The “plot_BSA()” function was used to draw the plot with a 5 Mb sliding window. (**A**–**C**) The scatter plots were exported from the BSA results, and the algorithms were ΔSNP, G-test, and ED. (**D**–**F**) The tricube-smoothed values of the corresponding statistics. The red line is the threshold set by the >85% quantile, and the blue line is the threshold set by *p*-values < 0.01.

**Figure 5:**
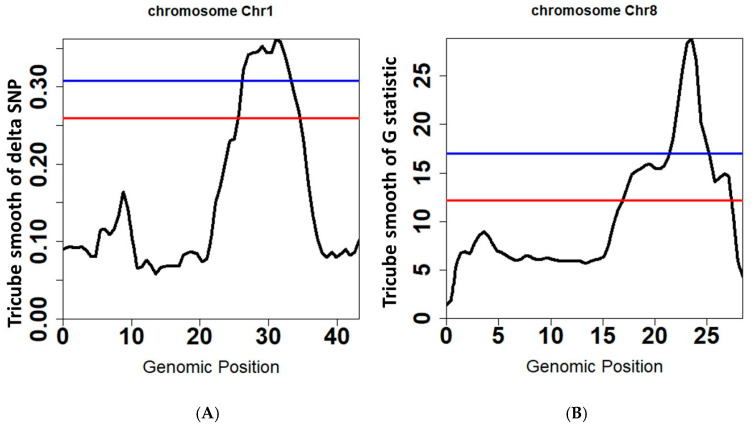
QTLs for rice seedling cold tolerance on chromosomes 1 and 8 identified by ΔSNP and G-test in PNGseqR plots drawn by the “plot_BSA()” function, and statistics are tricube-smoothed with a 5 Mb sliding window. (**A**) The line plot drawn by using the ΔSNP algorithm. (**B**) The line plot drawn by using the G-test algorithm. The red line is the threshold set by the >85% quantile and the blue line is the threshold set by *p*-values < 0.05.

To show that PNGSeqR can use DNA-seq data to map QTL, we reproduced the analysis described by Yang et al. [30] using PNGSeqR. Raw reads were downloaded from the NCBI database (accession number: SRP021494) and aligned to the v7 Nipponbare genome (http://rice.plantbiology.msu.edu/, accessed on 11 June 2022) using BWA-MEM v0.7.12 [31] with the default settings. SNPs were called following Best Practices [32,33] methods in GATK v3.7 [34] and exported as a data frame using the GATK “VariantsToTable” tool. We used the “BSA_filter()” function to import this data frame and to filter SNPs. Then, we used three algorithms to perform BSA and used *p*-value < 0.05 as a criterion for defining the thresholds. The analysis results were used to draw the scatter and line plots using “plot_BSA()” function (Figure 4). The QTLs on chromosomes 1 and 8 were significant when the *p*-value was lower than 0.05 for each algorithm (Figure 4, Table 3). However, the signals on chromosomes 2, 5, and 10 that were selected as QTLs in the published results [30] were not detected. When the fractile quantile was 85%, the QTLs on chromosomes 2, 5, and 10 were also detected (Figure 4, Table 3), indicating that the *p*-value < 0.05 threshold determined by the permutation test is much more stringent. In addition, PNGSeqR can select the chromosome(s) which contain QTL and plot the selected chromosome(s) (Figure 5).

### 2.3. Example 3—Mapping QTLs Using a RIL Population

**Figure 6:**
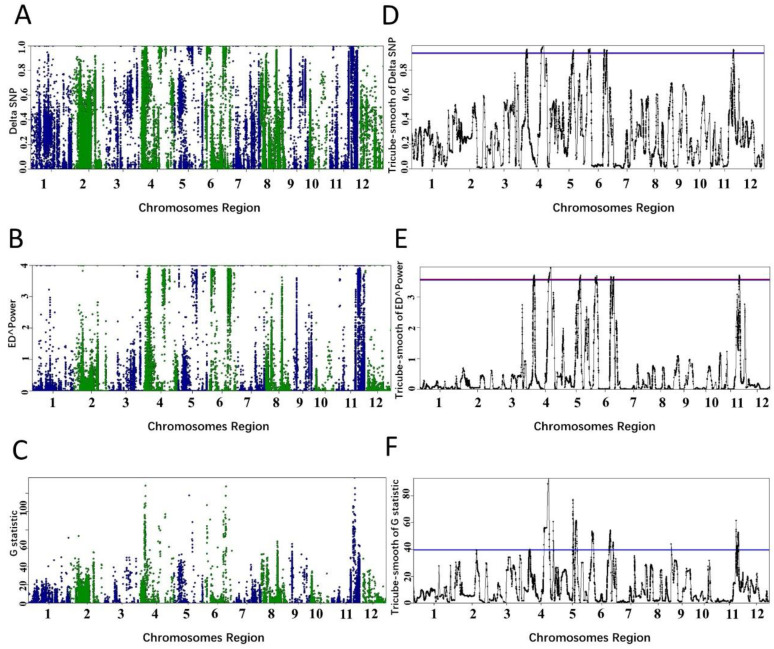
QTL identified by different algorithms in PNGseqR for rice cold tolerance in a RIL population. Plots were drawn by the “plot_BSA()” function with a 1 Mb sliding window. (**A**–**C**) The scatter plots were drawn by using the ΔSNP, Euclidean distance, and G-test algorithms. (**D**–**F**) Tricube-smoothed values on the genome. The red line is the threshold set by the >95% quantile and the blue line is the threshold set by *p*-values < 0.05.

In example 3, we demonstrate that PNGSeqR can handle bulk DNA-sequencing data of permanent populations. The data have been used to detect QTLs for rice cold tolerance [35]. Two DNA pools were constructed by selecting the top 20 cold-tolerant and cold-sensitive lines from an RIL population containing 190 lines. We downloaded the bulk DNA-sequencing data from NCBI (SRR6327817, SRR6327818) and aligned the data to the rice reference genome IRGSP-1.0_genome.fasta. SNP calling was performed as stated in example 2, producing a total of 1,386,657 SNPs. After filtering SNPs by running “BSA_filter()”, 147,494 SNPs were obtained and used to perform BSA. When *p*-value < 0.05, 6 QTLs on chromosomes 4, 4, 5, 6, 6, and 11 were detected by running “ED()” and “DeltaSNP()”, and 3 more QTLs were detected by running “Gprime()” (Figure 6 and Table 4). The lengths of candidate regions ranged from 8.81 to 9.81 Mb (Table 4). The QTLs on chromosome 11 detected in this study were not found by Sun et al. [35]. The lengths of candidate regions of QTLs detected by Sun et al. [35] were larger than those defined by the ED statistic, smaller than those defined by ΔSNP in this study, and similar to those defined by fractile quantile > 95% (Figure 6, Table S5). The functional gene LOC_Os06g39750 was in the candidate region on chromosome 6 detected by either statistic (Table 4). Results of the analysis indicate that PNGSeqR can handle bulk sequencing data of permanent populations.

Additionally, it is difficult to confirm candidate regions for unsmooth statistics in the three examples (Figures S1–S3). The tricube-smoothed statistics clearly identified the QTLs (Figure 2) and enabled users to define the candidate intervals. The comparison demonstrates that the tricube-smoothed statistics could remove noisy signals across the whole genome.

## 3. Discussion

Although tools for analyzing bulk sequencing data are publicly available [7,8,10,11], these tools have different shortcomings. For example, some tools could only analyze DNA or RNA data, some tools could not provide thresholds for declaring significant loci, and some tools could not prioritize candidate genes based on differential expression and gene annotation. To provide a user-friendly and all-in-one tool for performing BSA, we developed PNGSeqR, which provides four R functions to implement four algorithms. Theoretically, since the four algorithms are calculated based on the marker frequencies of two pools, any biparental segregation population could be used to perform BSA. As demonstrated in this study, PNGSeqR is not only applicable to temporary populations such as the F2 and F3 populations used in examples 1 and 2, but it is also suitable for permanent populations, such as the RIL population used in example 3.

For species that might not have high-quality genome sequences, there might be some errors in SNP data after performing SNP calling. “BSA_filter()” can eliminate low-quality or mismatched SNPs before analysis to ensure the accuracy of analysis. We tested the influence of the quality of the reference genome on BSA mapping results using the sequencing data in example 1. Maize B73 reference genome version 3 was assembled with second-generation sequencing technology [36] and the version 4 genome was assembled with the third-generation sequencing technology [37]. We found that there were no differences in the detection of the candidate region between the two reference genomes (Table S6, Figure S4), which is the same as the result in a rice study [38,39]. However, a high-quality reference genome might be desirable to ensure the mapping accuracy for highly heterozygous species, such as apple [9].

Tricube smoothing of each statistic integrated in PNGSeqR is crucial to map the correct candidate regions [7,9]. Tricube smoothing can remove the occasional extreme data that might be caused by low-quality reference genomes, and diminish the influence of these data [7]. However, since the size of the sliding window might be related to factors such as population type, sequencing depth, pool size, and impact mapping result [9,12,40], the size of the sliding window should be adjusted based on actual data.

We used RNA-seq data in example 1 to investigate whether the sequencing depth influenced the mapping region obtained by PNGSeqR. We randomly selected 1/5 and 1/2 reads from the 30× RNA-seq data, forming the 6× and 15× test datasets. A total of 29,310 SNPs and 56,943 SNPs were obtained from the 6× and 15× datasets and used for BSA. We found that the candidate region obtained by using the 6× dataset was larger than that obtained by using the 15× dataset and the full dataset (Table 1, Tables S7 and S8). Although a stable candidate region containing the target gene can be identified through these algorithms, the length of the candidate region generally increases with the decrease of the sequencing depth.

## 4. Materials and Methods

### 4.1. Data Import and Filtering

PNGSeqR uses a VCF file generated by GATK software as input. PNGSeqR can convert the VCF file into a data frame, in which the row indicates that the SNP information and columns are descriptive data. The first four columns indicate the chromosomes, position, reference alleles, and alternative alleles of the SNPs, respectively. Next, “AD.REF”, “AD.ALT”, “DP”, and “GQ” of SNPs of the two bulks are shown in the following eight columns, where “AD.REF” and “AD.ALT” indicate the read depth of the reference and alternative alleles in each bulk, “DP” indicates the coverage of SNPs of each bulk, and “GQ” indicates the quality control information of each bulk. If all the above columns are available, users can use PNGSeqR to perform subsequent analysis. To reduce noise and improve the quality of the result according to the users’ needs, the software offers options for SNP filtering based on total read depth, read depth in each bulk, the absolute differences in read depth between the bulks, and genotype quality score. The number of remaining SNP and removed SNPs can be printed when verbose is set as “True”.

### 4.2. Bulk Segregant Analyses

PNGSeqR provides four different BSA algorithms, which are optional according to the sequencing data, mainly including DNA-sequencing (DNA-seq) data and RNA-seq data. Among the four approaches, G-test [7], ΔSNP [11], and Euclidean distance (ED) [8] can be used for both DNA-seq data and RNA-seq data. Empirical Bayes [10] is the only algorithm that was suitable for RNA-seq data, and needs to provide the length of the reference genome and number of samples in each bulk. For more detailed information about these algorithms, please read the Supplementary Materials.

One major feature of PNGSeqR is that it provides a simple, efficient, and unified method to reduce the noise and estimate the threshold value based on different algorithms. To exclude the random noise generated from the variable sequence read coverage, we performed Nadaraya–Watson kernel regression [41,42], a local polynomial regression, for each BSA algorithm. The first application of tricube smoothing in BSA was performed to smooth the G statistic [7], then it was used to smooth ΔSNP [43] and ED statistics [9]. To obtain the candidate interval from BSA, PNGSeqR provides two methods to set threshold criteria for these four algorithms. One method is to sort the tricube-smoothed values and select the value at a specific quantile (95% or 99%) as the threshold [44,45]. The other method is to perform permutation tests and estimate the significance of each SNP based on the tricube-smoothed values [44,46]. PNGSeqR can export scatter and line plots containing the threshold line for each smooth algorithm.

### 4.3. DEG and GO Analyses

PNGSeqR can also integrate BSA with other strategies to predict and prioritize the causal genes [44,47]. Our shell script can calculate the read count of each RNA-seq sample, facilitating the users to perform differential expression analysis [31,48,49,50]. After that, DEG genes can also be used to perform GO analysis using PNGSeqR. These tools will help researchers to identify potential candidate genes within the candidate regions. The volcano plot showing the significance of DEGs could be printed and exported in PDF format, and the acyclic, bubble, and histogram plots showing the functional enrichment patterns could also be exported in PDF format after performing GO analysis. Lastly, a function was developed to identify the genes located in the candidate region, and whether these genes show differential expression.

## 5. Conclusions

PNGSeqR provides a user-friendly and convenient tool for researchers to integrate four major NGS-BSA algorithms. PNGSeqR can rapidly identify the candidate region by using tricube smoothing and the permutation test. The candidate region can be shortened by comparing the mapping results obtained using different algorithms. Based on the BSA mapping result, PNGSeqR can aid in prioritizing candidate genes through DEG and GO analysis.

## Figures and Tables

**Table 1:** The candidate region for maize small-kernel mutant identified by PNGseqR.

Criteria ^a^		Methods ^b^	Chrom ^c^	Start ^d^	End ^e^	Length ^f^
				——————Mb——————		
*p*-value	0.01	Bayes	8	0.08	11.28	11.20
		ED	8	0.08	10.65	10.57
		ΔSNP	8	0.08	10.00	9.92
		G-test	8	0.08	10.65	10.57
	0.005	Bayes	8	0.08	10.49	10.41
		ED	8	0.08	10.20	10.12
		ΔSNP	8	0.08	9.36	9.28
		G-test	8	0.08	8.05	7.97
	0.001	Bayes	8	1.46	3.42	1.96
		ED	8	0.08	3.37	3.30
		ΔSNP	8	0.08	2.21	2.13
		G-test	8	0.08	1.67	1.59
Quantile	99%	Bayes	8	0.08	11.28	11.20
		ED	8	0.08	10.65	10.57
		ΔSNP	8	0.08	10.00	9.92
		G-test	8	0.08	10.65	10.57
	99.5%	Bayes	8	0.08	11.03	10.95
		ED	8	0.08	10.65	10.57
		ΔSNP	8	0.08	9.36	9.28
		G-test	8	0.08	10.45	10.37
	99.9%	Bayes	8	1.46	3.42	1.96
		ED	8	0.08	3.29	3.21
		ΔSNP	8	0.08	3.29	3.21
		G-test	8	0.08	3.29	3.21

Notes: ^a^: Multiple thresholds were used to declare the candidate region by defining *p*-value and quantile. ^b^: The statistics used to identify the candidate region. ^c^: The chromosome that contains the candidate region. ^d^: The start position of the candidate region. ^e^: The end position of the candidate region. ^f^: The length of the candidate region.

**Table 2:** The differential expression genes in the candidate region for the maize small-kernel mutant.

Chrom ^a^	Start ^b^	End ^c^	Gene ^d^	Log_2_ (foldchange) ^e^	FDR ^f^
8	563764	569489	Zm00001d008179	−1.6552	4.96 × 10^−3^
8	909115	910111	Zm00001d008196	−7.1323	1.86 × 10^−3^
8	957005	966468	Zm00001d008198	2.4286	3.83 × 10^−2^
8	1238180	1242266	Zm00001d008209	2.6675	4.95 × 10^−5^
8	1299092	1300668	Zm00001d008211	−3.7562	6.48 × 10^−4^
8	1983066	1984497	Zm00001d008229	−2.6535	3.86 × 10^−2^
8	3193712	3222186	Zm00001d008256 ^g^	−1.5896	6.63 × 10^−13^

Notes: ^a^: The chromosome. ^b^: The start position of candidate genes. ^c^: The end position of candidate genes. ^d^: The gene IDs. ^e^: The logarithm of fold changes. ^f^: False discovery rate. ^g^: The target gene verified by Chen et al. (2021) [20].

**Table 3:** QTLs for cold tolerance at the seedling stage in rice identified by different algorithms in PNGseqR.

Chrom ^a^	ΔSNP (Mb) ^b^		G-Test (Mb) ^c^		ED (Mb) ^d^	
	Quantile > 85%	*p*-Value < 0.05	Quantile > 85%	*p*-Value < 0.05	Quantile > 85%	*p*-Value < 0.05
Chr1	25.61–34.62	26.20–33.34	25.56–34.90	26.22–33.63	25.45–34.49	26.12–33.20
Chr2	9.58–19.44	10.06–10.60	9.58–19.58	10.01–10.51	9.28–19.64	9.73–10.40
Chr5	27.09–28.75	-	27.35–28.68	-	27.14–29.96	-
Chr8	17.04–27.30	21.27–25.24	16.92–27.37	21.40–25.17	16.90–27.54	21.34–25.43
Chr10	17.50–20.25	-	17.67–20.26	-	17.66–20.33	-

Notes: ^a^: The chromosomes containing the candidate regions. ^b^: The candidate regions identified by the ΔSNP algorithm. ^c^: The candidate regions identified by the G-test algorithm. ^d^: The candidate regions identified by the Euclidean distance algorithm.

**Table 4:** QTLs detected for cold tolerance in a rice RIL population using different statistics in PNGseqR.

Chrom ^a^	ED (Mb) ^b^			ΔSNP (Mb) ^c^			G-Test (Mb) ^d^		
	Start	End	Size	Start	End	Size	Start	End	Size
4	5.53	6.30	0.77	5.57	6.32	0.75	5.98	6.19	0.21
4	21.31	23.69	2.38	21.03	23.69	2.66	20.81	23.69	2.88
5	-	-	-	-	-	-	15.31	16.07	0.77
5	18.97	19.57	0.60	19.18	19.57	0.39	18.07	19.21	1.14
6	5.37	6.99	1.62	5.32	7.00	1.68	5.23	7.00	1.77
6	21.79	24.68	2.89	21.79	24.68	2.89	23.31	24.80	1.49
6	-	-	-	-	-	-	27.21	27.55	0.34
11	-	-	-	-	-	-	21.71	21.98	0.27
11	23.89	24.44	0.55	23.92	24.44	0.52	23.37	24.30	0.93
Total	-	-	8.81	-	-	8.89	-	-	9.81

Notes: For each statistic, the threshold for identifying QTL is *p*-value < 0.05. ^a^: The chromosomes containing the candidate regions. ^b^: The candidate regions identified by Euclidean distance in PNGseqR: start, end, and size indicate the start position, end position, and size of the candidate regions, respectively. ^c^: The candidate regions identified by ΔSNP in PNGseqR. ^d^: The candidate regions identified by the G-test in PNGseqR.

## Data Availability

Sample data were deposited at: https://github.com/smilejeen/PNGseqR, accessed on 30 June 2022.

## Supplementary Materials

The following supporting information can be downloaded at: https://www.mdpi.com/article/10.3390/plants11141821/s1. Supplementary Figure S1: The distribution of unsmooth statistics for example 1 across the whole genome. Supplementary Figure S2: The distribution of unsmooth statistics for example 2 across the whole genome. Supplementary Figure S3: The distribution of unsmooth statistics for example 3 across the whole genome. Supplementary Figure S4: Positioning results by PNGseqR for maize small kernel mutant with version3 maize B73 reference genome Supplementary Table S1: Description of PNGseqR functions and arguments. Supplementary Table S2. The list of differential expression genes identified in example1. Supplementary Table S3. The gene IDs and GO terms of maize genes in B73 reference genome version 4. Supplementary Table S4. The list of genes in the candidate region on chromosome 8. Supplementary Table S5. QTLs detected for cold-tolerance in a rice RIL population by PNGseqR. Supplementary Table S6. The candidate region for maize small kernel mutant identified by PNGseqR (based on B73 version3 reference genome). Supplementary Table S7. The candidate region for maize small kernel mutant identified by PNGseqR (using 1/5 RNA-seq reads). Supplementary Table S8. The candidate region for maize small kernel mutant identified by PNGseqR (using 1/2 RNA-seq reads). Supplementary Implementation.
