# Supplementary material for: PNGSeqR: An R Package for Rapid Candidate Gene Selection through Pooled Next-Generation Sequencing
Source: Plants (Basel). 2022 Jul 11;11(14):1821. doi: 10.3390/plants11141821 (PMC9315718; doi:10.3390/plants11141821)
Supplement: Supplementary file 1 [file plants-11-01821-s001.zip › FigS1-4.pdf]

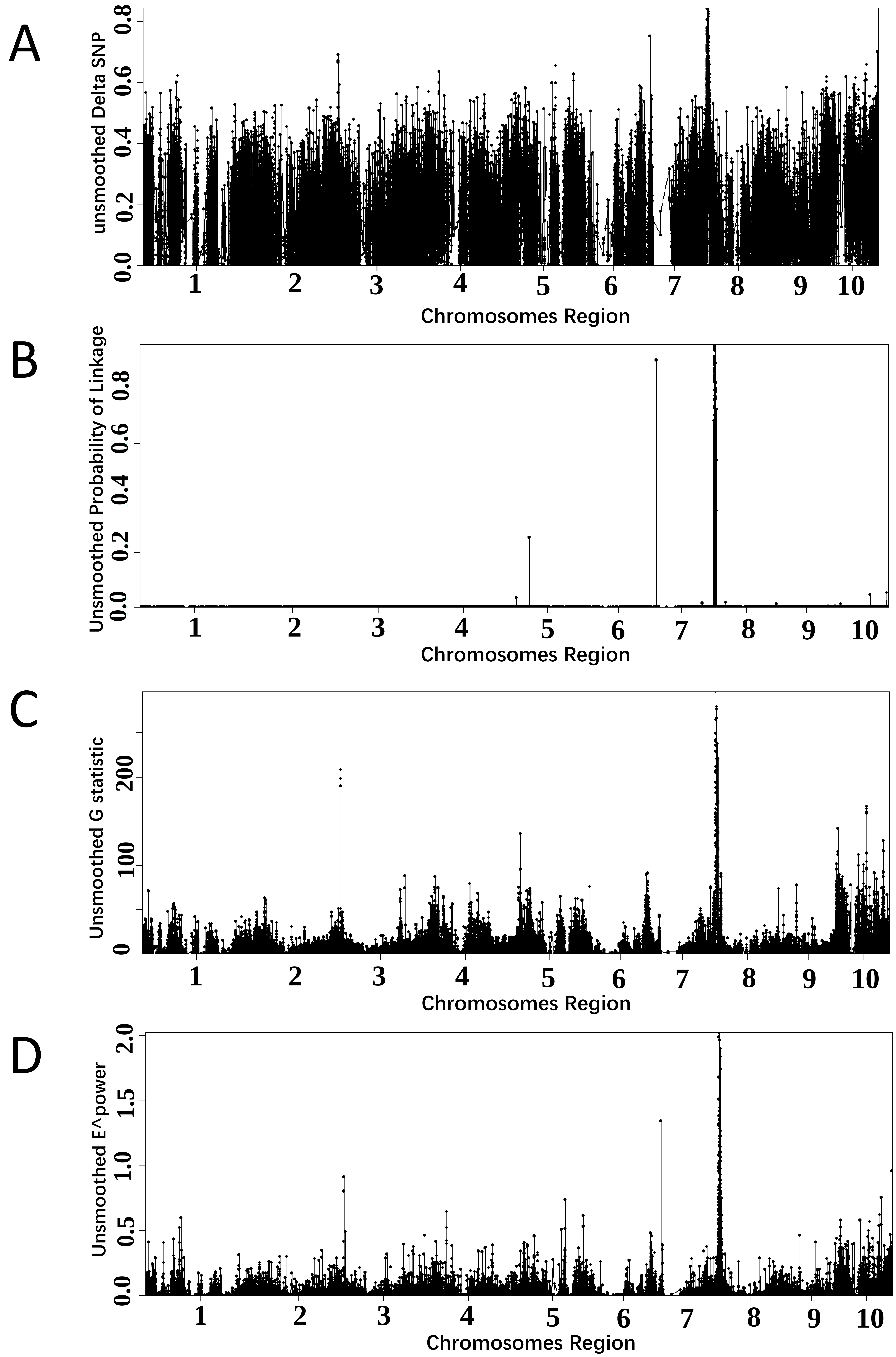

Figure S1. The distribution of unsmooth statistics for example 1 across the whole genome. A–D indicate the plots of unsmoothed ΔSNP, Bayes, G-test, and ED algorithms, respectively.

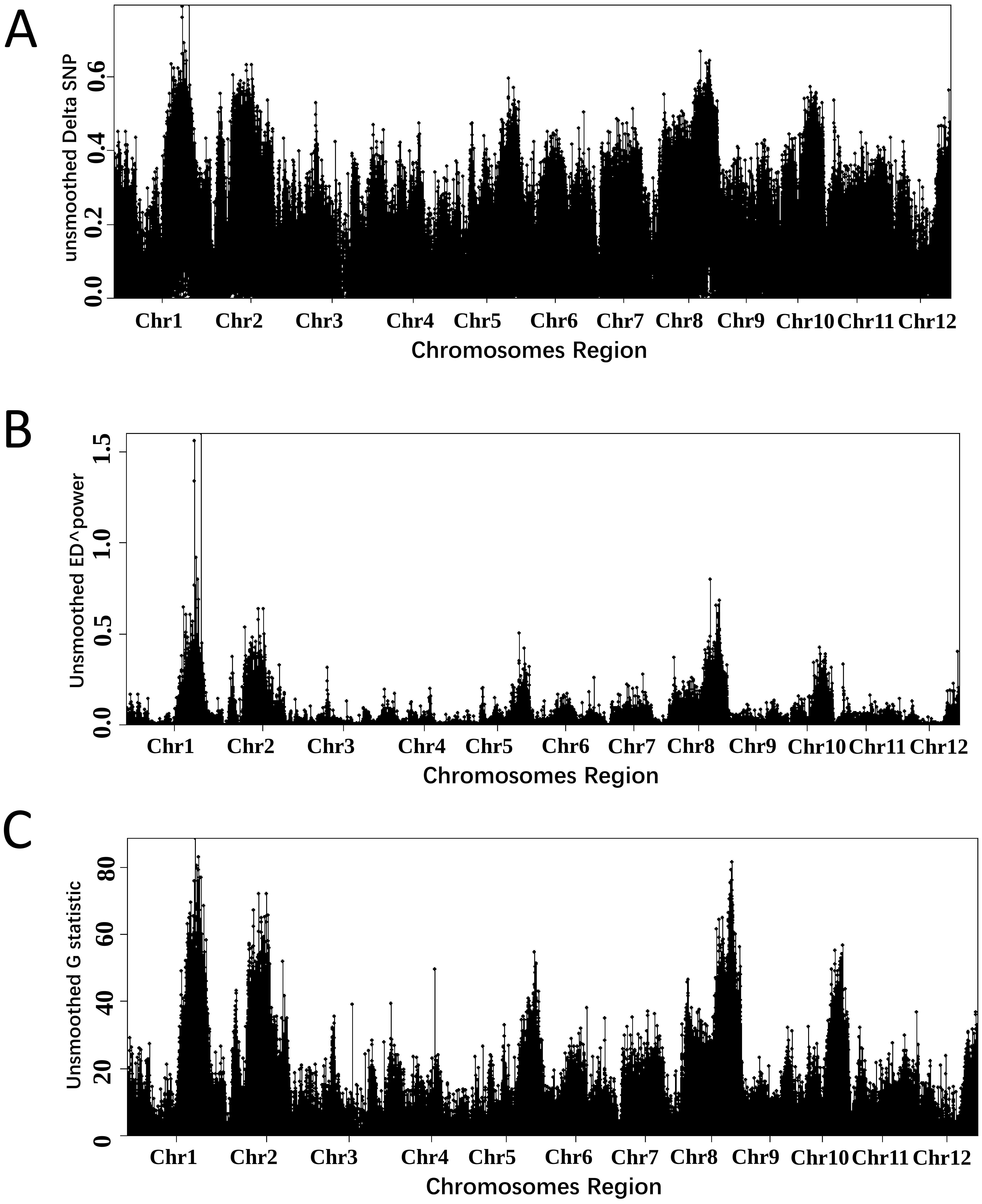

Figure S2. The distribution of unsmooth statistics for example 2 across the whole genome. A–D indicate the plots of unsmoothed ΔSNP, ED, and G-test algorithms, respectively.

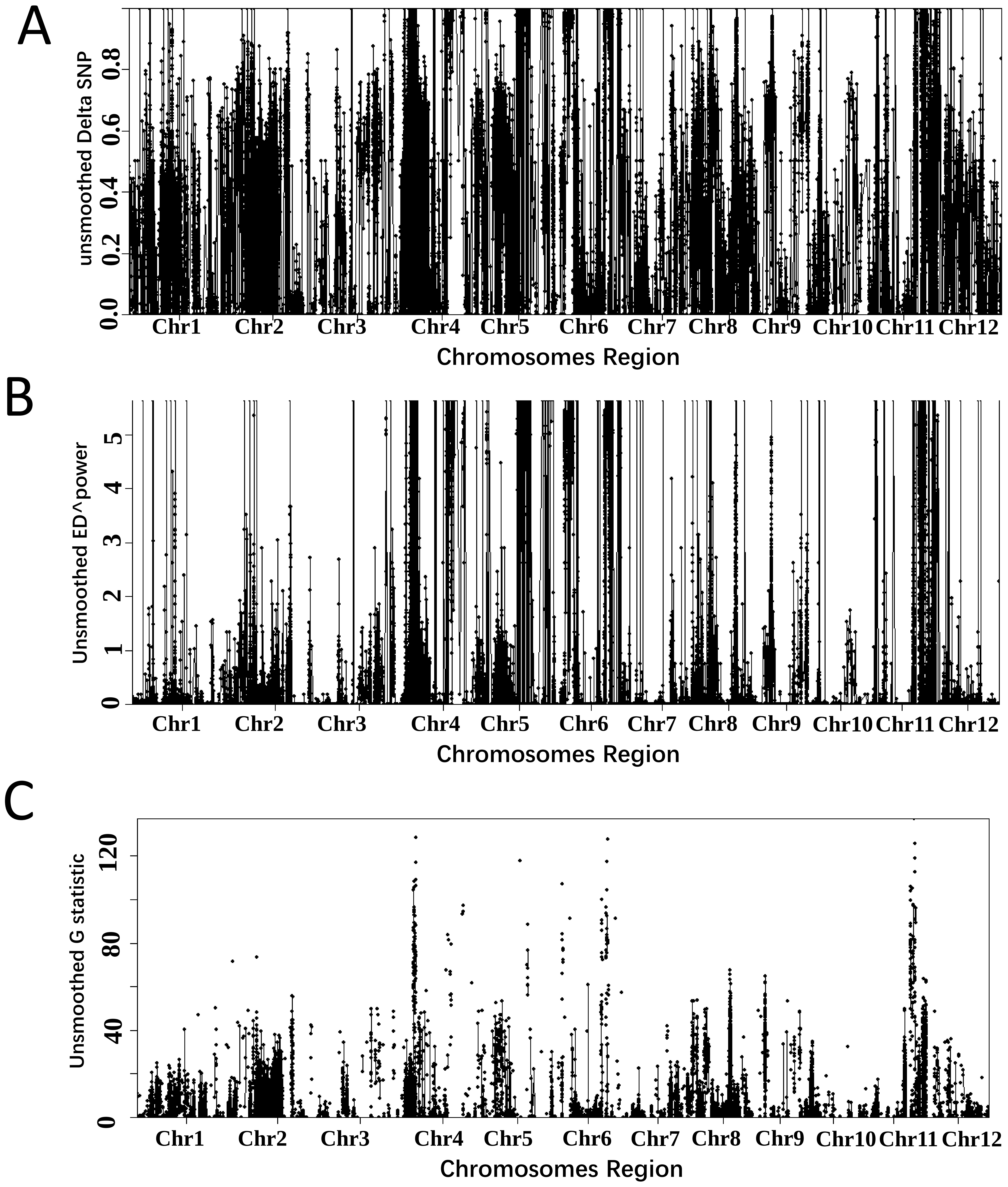

Figure S3. The distribution of unsmooth statistics for example 3 across the whole genome. A–D indicate the plots of unsmoothed ΔSNP, ED, and G-test algorithms, respectively.

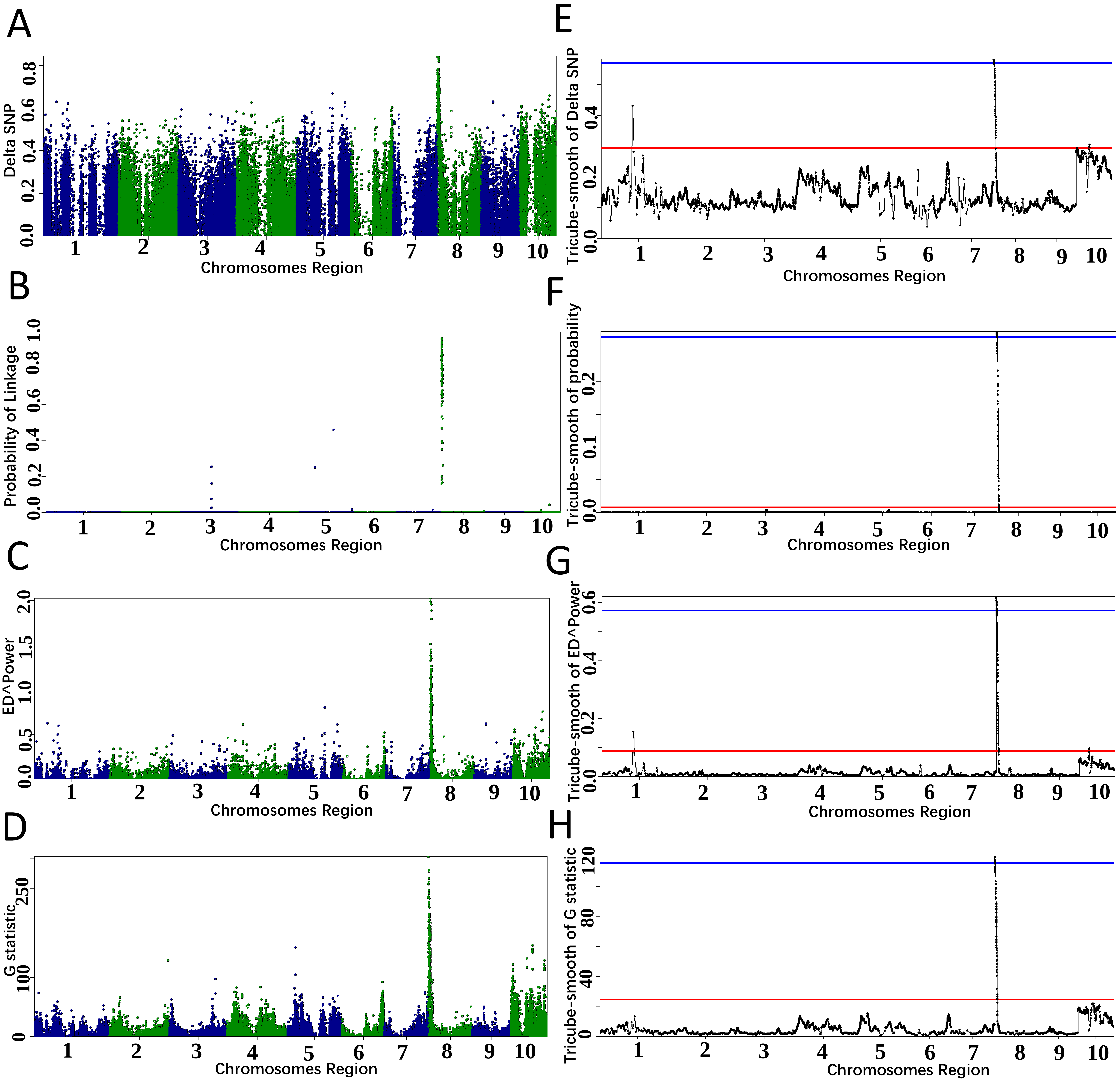

Figure S4. Positioning results by PNGseqR for maize small kernel mutant with version3 maize B73 reference genome. Plots produced by the "plot_BSA()" function with a 5 Mb sliding window: (A–D).The scatter plots exported from the BSA results, the used algorithms are ΔSNP, Bayes, ED, and G-test. (E–H) Tricube-smoothed values on the genome. The red line threshold shows the region containing SNPs whose tricube-smoothed values are higher than 99.5% of all values, and the blue line threshold shows the SNPs whose *P-values* are lower than 0.001 based on permutation test.
