# Supplementary material for: PNGSeqR: An R Package for Rapid Candidate Gene Selection through Pooled Next-Generation Sequencing
Source: Plants (Basel). 2022 Jul 11;11(14):1821. doi: 10.3390/plants11141821 (PMC9315718; doi:10.3390/plants11141821)
Supplement: Supplementary file 1 [file plants-11-01821-s001.zip › Supplementary material.pdf]

Supplementary material

Implementation

PNGSeqR is an R package developed to perform Next generation sequencing Bulk Segregant Analysis (NGS-BSA). The package import NGS-SNP in VCF format, can map genetic loci controlling both quantitative and qualitative trait with four algorithms, including G-test[1] , empirical Bayes[2] , ΔSNP[3] , and Euclidean distance (ED)[4] , and can prioritize the causal genes by combining with DEG and GO analysis.

SNP Calling for DNA sequencing data:

We provide linux shell commands to get input files in Variant Call Format (VCF) format from DNA sequencing data for PNGseqR. The reference genome should be downloaded before running the shell commands. Several software needs to be downloaded and installed in advance, such as BWA[5], SAMtools[6], Picard (http://broadinstitute.github.io/picard) and GATK (https://github.com/broadinstitute/gatk/releases). First, the reference genome must be indexed following the command lines:

bwa index -a ${reference genome}
samtools faidx ${reference genome}
java -jar ${picardpath}/CreateSequenceDictionary.jar REFERENCE=${reference genome} OUTPUT= ${reference genome}.dict

Then, **BWA** software is used to map sequencing reads to the reference genome following the command line:

bwa mem -t 4 -M -R ${reference genome} ${fasta1} ${fasta2} > ${names}.sam

Then **Picard** software is used to sort file and convert it into BAM format following the command

line:

```
java -jar ${picardpath}/SortSam.jar VALIDATION_STRINGENCY=SILENT INPUT=${names}.sam OUTPUT=${names}.sorted.bam SORT_ORDER=coordinate
```

Picard is used to obtain unique reads, fix mate information, and add read group information, then **SAMtools** is used to establish sequence indexes, the command lines are as follows:

```
java -jar picard.jar CleanSam INPUT=${names}.sorted.bam OUTPUT=${names}.sorted.clean.bam

java -jar picard.jar FixMateInformation INPUT=${names}.sorted.clean.bam OUTPUT=${names}.sorted.clean.fixed.bam SO=coordinate

java -jar picard.jar AddOrReplaceReadGroups INPUT=${names}.sorted.clean.fixed.bam OUTPUT=${names}.sorted.clean.fixed.group.bam LB=WH SO=coordinate RGPL=ILLUMINA PU=barcode SM=WH

samtools index ${names}.sorted.clean.fixed.group.bam

java -jar picard.jar MarkDuplicatesWithMateCigar INPUT=${names}.sorted.clean.fixed.group.bam OUTPUT=${names}.sorted.clean.fixed.group_DEDUP.bam M=${names}.sorted.clean.fixed.group_DEDUP.mx AS=true REMOVE_DUPLICATES=true MINIMUM_DISTANCE=500, TMP_DIR=${tmp}

samtools index ${names}.sorted.clean.fixed.group_DEDUP.bam
```

At last, **GATK** software is used to perform SNP calling, the command lines are:

```
java -jar ${gatkpath} HaplotypeCaller -R ${reference genome} -I ${names}.sorted.clean.fixed.group_DEDUP.bam -O ${output path}/${names}.gvcf

java -jar ${gatkpath} CombineGVCFs -R ${reference genome} -V ${output path}/${bulk1name} -V ${output path}/${bulk2name} -O ${bulk1name}_${bulk2name}.gvcf

java -jar ${gatkpath} GenotypeGVCFs -R ${reference genome} --variant ${bulk1name}_${bulk2name}.gvcf -O ${bulk1name}_${bulk2name}.vcf

java -jar ${gatkpath} SelectVariants -R ${reference genome} -select 'QD > 2.0' -select 'FS <
```

40.0' -select 'MQRankSum > -12.5' -select 'ReadPosRankSum > -8.0' -select 'DP > 20.0' -V ${bulk1name}_${bulk2name}.vcf -O ${bulk1name}_${bulk2name}.filter.vcf

After running SNP calling, the input file for PNGseqR will be outputted in VCF format.

SNP Calling for RNA sequencing data:

The linux shell commands to get input files in VCF format for PNGseqR are also provided. The shell commands for RNA sequencing data have a little different from those prepared for DNA sequencing data. In addition to using SAMtools[6], Picard (http://broadinstitute.github.io/picard) and GATK (https://github.com/broadinstitute/gatk/releases), Hisat2 (https://github.com/DaehwanKimLab/hisat2) is used to handle RNA sequencing reads.

Firstly, **hisat2** is used to mapping RNA sequencing data to reference genome, producing files in SAM format. The command line for paried-end sequencing data is:

${hisat2path}/hisat2 -x ${hisat2 index} -1 ${fasta1} -2 ${fasta2} -S ${output path}/${names}.sam

The command line for paried-end sequencing data is:

${hisat2path}/hisat2 -x ${hisat2 index} -U ${fasta} -S ${output path}/${names}.sam

Users need to create HISAT2 index from reference genome as follow:

${hisat2path}/hisat2-build -f ${reference genome} ${hisat2 index}

Then, **Samtools** is used to convert files in SAM format to BAM format, and sort reads by chromosomes. The output files are in BAM format. Only unique mapping reads are allowed for further analysis. The command lines are:

samtools view -h -q 50 ${output path}/${names}.sam | samtools view -bS -> ${output

path}/unique.${names}.bam

samtools sort ${output path}/unique.${names}.bam ${output path}/unique.${names}.sort

samtools index ${output path}/unique.${names}.sort.bam

Then **picard** software is used to add groups for BAM files as follows:

java -jar ${picardpath}/AddOrReplaceReadGroups.jar I=${output path}/unique.${names}.sort.bam O=${output path}/unique.${names}.GR.bam RGID=unique.${names} RGLB=A RGPL=illumina RGPU=nounit RGSM=unique.${names}

At last, **GATK** software is used to perform SNP calling

java -jar ${gatkpath} -T UnifiedGenotyper -R ${reference genome} -I ${output path}/unique.sort.${bulk1name}.GR.bam -I ${output path}/unique.sort.${bulk2name}.GR.bam --heterozygosity 0.1 -stand_call_conf 50.0 -stand_emit_conf 20.0 -glm BOTH --num_threads 2 -ploidy 2 -U ALLOW_N_CIGAR_READS -o ${output path}/${bulk1name}_${bulk2name}_snp.vcf

Normally, the SNPs in the VCF file need to be filtered based on the quality of SNPs, the command line is:

java -jar ${gatkpath} -T SelectVariants -R ${reference genome} --variant ${output path}/${bulk1name}_${bulk2name}_snp.vcf -select 'QD > 2.0' -select 'FS < 40.0' -select 'MQRankSum > -12.5' -select 'ReadPosRankSum > -8.0' -select 'DP > 20.0' --restrictAllelesTo BIALLELIC --selectTypeToInclude SNP --sample_name unique.${bulk1name} --sample_name unique.${bulk2name} --excludeFiltered --excludeNonVariants -o ${output path}/${bulk1name}_${bulk2name}_snp.filt.vcf

Count reads for RNA-seq data:

PNGseqR can perform differential expression analysis, which requires users to prepare reads count file. DEseq2 is the dependent package in this step. Here, we provide a command to calculate reads count file. This command relies on Htseq-count[7] software, and need mapping file in BAM format and reference genome in GTF format. The command line is:[1]

htseq-count ${output path}/unique.${names}.sort.bam -f bam ${GTF path} > ${output path}/${names}.RC.txt

The reads count file containing reads mapped to genes in each sample is produced in this step, and can be used as the input file for differential expression analysis.

Empirical Bayesian algorithm:

The conditional probability is defined by the equation (Liu et al. 2012):

$$P(noR|X_1,X_2) = \frac{P(X_1,X_2|noR)P(noR)}{P(X_1,X_2|noR)P(noR) + P(X_1,X_2|R)P(R)}$$

$P(noR)$ is the prior probability that there is no recombination between a randomly selected SNP and the causal gene in the mutant pool. In contrast, $P(R)$ is the probability that there is recombination. X1 and X2 indicate the counts of SNP alleles in the mutant pool. Supposing d is the genetic distance (centimorgans) between a randomly-selected SNP and the causal gene, we can use Haldane's mapping function to compute the prior probability that there is no recombination between the SNP and the causal gene. The function is:

$$q(d) = \{1 - \frac{1}{2}[1 - exp(-2d)]\}^{2N},$$

where N denotes the number of plants in the mutant pool.

For each SNP:

$$P\left(pm \le \frac{1}{2} \middle| w_{m,} w\right) = \frac{P\left(w_{m,} w \middle| pm \le \frac{1}{2}\right) P\left(pm \le \frac{1}{2}\right)}{P(wm, w)},$$

where pm is the proportion of a mutant allele at the SNP in the wild type pool w_m. SNPs that have high values of both $P(noR|x_1, x_2)$ and $P\left(pm \le \frac{1}{2} \middle| w_{m,} w\right)$ are in close linkage with the causal gene.

ED power algorithm:

The algorithm ED was first used in MMAPPR (Hill et al. 2013), which used the RNA-Seq data of two pools to perform BSA analysis, and didn't require parental strain information:

$$ED = \sqrt{(A_{mut} - A_{wt})^2 + (C_{mut} - C_{wt})^2 + (T_{mut} - T_{wt})^2 + (G_{mut} - G_{wt})^2},$$

where each letter (A, C, G, T) corresponds to the frequency of a SNP allele in either the mutant pool or the wild type pool. In practical experiment, the users need to raise ED to several powers (ED^x) to decrease noise (such as ED^4). Moreover, this algorithm is applicable to BSA analysis that uses both RNA-seq and DNA-Seq data.

G' algorithm:

G is defined by the equation (Magwene et al. 2011):

$$G = 2 * \sum_{i=1}^{4} n_i * ln\left(\frac{obs(n_i)}{exp(n_i)}\right)$$

For each SNP, n_i indicates the depths of the reference and alternate alleles in each pool, and i ranges from 1 to 4 (Table2). $obs(n_i)$ and $exp(n_i)$ indicate the observed and expected depth of each allele, respectively. Assuming read depth is equal for all alleles, $exp(n_i)$ could be calculated as follows:

$$exp(n1) = \frac{(n1 + n2) * (n1 + n3)}{(n1 + n2 + n3 + n4)}$$

$$exp(n2) = \frac{(n2 + n1) * (n2 + n4)}{(n1 + n2 + n3 + n4)}$$

$$exp(n3) = \frac{(n3 + n1) * (n3 + n4)}{(n1 + n2 + n3 + n4)}$$

$$exp(n4) = \frac{(n4 + n2) * (n4 + n3)}{(n1 + n2 + n3 + n4)}$$

Table2: Per SNP allele frequency table, adapted from Magwene et al. (2011)

Allele	High Bulk	Low Bulk
Reference	n1	n2
Alternate	n3	n4

Δ(SNP-index) algorithm:

Δ(SNP-index) is the difference of SNP frequencies (SNP-index) between two pools (Takagi et al. 2013), the SNP-index in each pool is calculated as:

$$SNP\text{-}index_{each\ bulk} = \frac{Alternate\ allele\ depth}{Total\ read\ depth},$$

and Δ(SNP-index) is calculated as:

$$\Delta(SNP - index) = SNP\text{-}index_{bulk1} - SNP\text{-}index_{bulk2}.$$

G’ and Δ(SNP-index) algorithms are applicable for both DNA-seq and RNA-seq data.

Tricubed-smoothed analyses

Nadaraya-Watson kernel regression, a local polynomial regression approach, is used for smoothing discontinuous variables in PNGseqR [8]; Watson, 1964). This smoothing method gives larger weight to the statistics of markers that are close to the focal marker. The statistics of markers are calculated by using the ***locfit()*** function of the **locfit** package, and the window size should be defined in this step. The Tricube weighting values of all SNPs, k_j, are calculated. Within the window *W*:

$$k_j = \frac{(1 - D_j^3)^3}{\sum_{j\ in\ W}(1 - D_j^3)^3},$$

where D is the distance of SNP j from the focal SNP:

$$D_j = \frac{distance\ of\ j\ from\ focal\ SNP}{window\ size},$$

within a defined window, the parameter D that requires smoothing is multiplied by weighting value k_j for each SNP.

Reference

1. Magwene, P.M.; Willis, J.H.; Kelly, J.K. The Statistics of Bulk Segregant Analysis Using Next Generation Sequencing. *PLoS Computational Biology*. **2011**, *7*, e1002255, doi:10.1371/journal.pcbi.1002255.
2. Liu, Sanzhen and Yeh, Cheng Ting and Tang, Ho Man and Nettleton, Dan and Schnable, Patrick S. Gene Mapping via Bulked Segregant RNA-Seq (BSR-Seq). *PLoS ONE*. **2012**, *7(5)*, e36406, 10.1371/journal.pone.0036406.
3. Takagi, H.; Abe, A.; Yoshida, K.; Kosugi, S.; Natsume, S.; Mitsuoka, C.; Uemura, A.; Utsushi, H.; Tamiru, M.; Takuno, S.; et al. QTL-Seq: Rapid Mapping of Quantitative Trait Loci in Rice by Whole Genome Resequencing of DNA from Two Bulked Populations. *The Plant Journal*. **2013**, *74*, 174–183, doi:10.1111/tpj.12105.
4. Hill, J.T.; Demarest, B.L.; Bisgrove, B.W.; Gorsi, B.; Su, Y.-C.; Yost, H.J. MMAPPR: Mutation Mapping Analysis Pipeline for Pooled RNA-Seq. *Genome Research*. **2013**, *23*, 687–697, doi:10.1101/gr.146936.112.
5. Li, H.; Durbin, R. Fast and Accurate Short Read Alignment with Burrows-Wheeler Transform. *Bioinformatics*. **2009**, *25*, 1754–1760, doi:10.1093/bioinformatics/btp324.
6. Danecek, P.; Bonfield, J.K.; Liddle, J.; Marshall, J.; Ohan, V.; Pollard, M.O.; Whitwham, A.; Keane, T.; McCarthy, S.A.; Davies, R.M.; et al. Twelve Years of SAMtools and BCFtools. *GigaScience*. **2021**, *10*, doi:10.1093/gigascience/giab008.
7. Putri, G.H.; Anders, S.; Pyl, P.T.; Pimanda, J.E.; Zanini, F. Analysing High-Throughput Sequencing Data in Python with HTSeq 2.0. *Bioinformatics*. **2022**, btac166, doi:10.1093/bioinformatics/btac166.
8. Nadaraya, E.A. On Estimating Regression. *Theory of Probability Its Applications*. **1964**, *9*, 141–142, doi:10.1137/1109020.
